# Acetanilide

**Published:** 1890-02-08

**Authors:** 


### THERAPEUTICS.

### ACETANILIDE.

"The treatment of enteric fever by acetanilide" is the title of a paper by Dr. W. Peirce, of the Coast Hospital, Sydney (*Practitioner*, January, 1890). Dr. Peirce remarks that economy of vital energy appears to be the broad basis on which the treatment of enteric fever should rest . . . . to attain this, rest and reduction of heat are indispensable . . . . in acetanilide (which, by-the-bye, is a synonym for antifebrin) we possess a medicine which, by influencing metabolism, enables us to reduce temperature with certainty, rapidity, and safety. . . . the power of the nervous system being damaged under the influence of the febrile poison, excessive heat becomes generated. . . . acetanilide, by restoring this deteriorated nerve power, prevents high temperature, and, moreover, induces perspiration. Now we believe that too much importance has been attached to the reduction of temperature in fevers. Admitting that high temperature alone may be fatal, on the other hand a certain amount of high temperature must occur, for with it the metabolism is associated by which *materies morbi* is eliminated. Dr. Peirce seems to recognize this, for he says if the medicine is given so frequently as to keep temperature at a normal rate, "a very undesirable condition is apt to be induced, needing prolonged stimulation and the application of external heat." He also mentions that the long duration of some of his cases seems to indicate that the suppression of temperature retarded a salutary elimination by heat of the febrile *materies morbi*. "It may be that sometimes the disease is increased in duration; its intensity, however, being toned down and beneficially spread over a wider area." With the first part of this sentence we fully agree, but we question the second portion. The whole subject of the influence of remedies which tend to reduce the heat of the body demands attentive investigation. At present, by the use of such remedies, we combat a symptom. And we certainly thereby do not succeed in shortening the malady.

